# Dual spindles assemble in bovine zygotes despite the presence of paternal centrosomes

**DOI:** 10.1083/jcb.202010106

**Published:** 2021-09-22

**Authors:** Isabell Schneider, Marta de Ruijter-Villani, M. Julius Hossain, Tom A.E. Stout, Jan Ellenberg

**Affiliations:** 1 Cell Biology and Biophysics Unit, European Molecular Biology Laboratory, Heidelberg, Germany; 2 Department of Clinical Sciences, Faculty of Veterinary Medicine, Utrecht University, Utrecht, the Netherlands; 3 Division of Woman and Baby, University Medical Centre Utrecht, Utrecht, the Netherlands

## Abstract

The first mitosis of the mammalian embryo must partition the parental genomes contained in two pronuclei. In rodent zygotes, sperm centrosomes are degraded, and instead, acentriolar microtubule organizing centers and microtubule self-organization guide the assembly of two separate spindles around the genomes. In nonrodent mammals, including human or bovine, centrosomes are inherited from the sperm and have been widely assumed to be active. Whether nonrodent zygotes assemble a single centrosomal spindle around both genomes or follow the dual spindle self-assembly pathway is unclear. To address this, we investigated spindle assembly in bovine zygotes by systematic immunofluorescence and real-time light-sheet microscopy. We show that two independent spindles form despite the presence of centrosomes, which had little effect on spindle structure and were only loosely connected to the two spindles. We conclude that the dual spindle assembly pathway is conserved in nonrodent mammals. This could explain whole parental genome loss frequently observed in blastomeres of human IVF embryos.

## Teaser

Schneider and de Ruijter-Villani et al. demonstrate that two spindles assemble around the parental genomes in bovine zygotes even though, like human zygotes, they inherit centrioles from the sperm. The two independent microtubule arrays form by self-organization with only loosely connected centrosomes.

## Introduction

Mammalian fertilization involves the fusion of a sperm cell with an oocyte to give rise to a totipotent zygote, from which a whole new organism can develop. This development begins with the first mitotic cell divisions. One would expect that these divisions are highly controlled, but they are surprisingly prone to chromosome mis-segregations. Resulting postzygotic or “mosaic” aneuploidy, where a subset of cells in the embryo has an aberrant number of chromosomes, is frequently observed in human preimplantation embryos from parents seeking assisted reproduction treatments (van Echten-Arends et al., 2011; McCoy et al., 2015; Wells and Delhanty, 2000; Daphnis et al., 2008; Vanneste et al., 2009; Mertzanidou et al., 2013). A similar frequency of aneuploidy and other chromosomal aberrations is expected to occur in preimplantation-stage embryos after natural conception, as ∼60% of conceptions are lost early on (Macklon et al., 2002). A high incidence of aneuploidy within an embryo is acknowledged to be a major cause of developmental failure and early pregnancy loss. A recent study has indicated a particularly strong negative selection against postzygotic aneuploidies before day 5 of development, while aneuploidies of meiotic origin seem to frequently propagate further and are thus more often observed upon pregnancy loss at later stages (McCoy et al., 2015). The frequent occurrence of postzygotic aneuploidy and early embryonic mosaicism is a major obstacle for embryo assessment after in vitro fertilization (IVF) in fertility clinics (Taylor et al., 2014; Munné et al., 2017; Fragouli et al., 2017; Vera-Rodriguez and Rubio, 2017).

A similarly high degree of postzygotic aneuploidy as in human embryos has also been reported in porcine, nonhuman primate, murine, bovine, and equine embryos, suggesting that this phenomenon is common in the preimplantation development of many mammalian species (Zudova et al., 2003; Dupont et al., 2010; Bolton et al., 2016; Tšuiko et al., 2017; Shilton et al., 2020). Despite the widespread occurrence and often severe developmental consequences of postzygotic aneuploidy, we do not yet understand why the critical cell divisions at the beginning of mammalian life are so prone to errors, especially due to limited access to the relevant samples and technological difficulties to visualize these events in live mammalian embryos.

The first division of the embryo is an exceptional mitosis. After fertilization, the parental genomes are replicated within the two separate pronuclei (PNi). Upon entry into mitosis, the nuclear envelopes (NEs) break down and the two spatially separated sets of parental chromosomes must interact in a coordinated fashion with the assembling mitotic apparatus of the zygote to allow synchronous and faithful segregation into two daughter cells. It was long assumed that the parental genomes would mix immediately after NE breakdown (NEBD) and subsequently be segregated using a single zygotic spindle. In fact, even the definition of when a fertilized oocyte becomes a human embryo is based on the time when the parental genomes merge in some legal systems (e.g., Germany, § 8 Abs. 1, Embryonenschutzgesetz). However, using high-resolution imaging of live embryos by light-sheet microscopy, we recently showed that in mouse zygotes, two separate microtubule arrays form around each of the two parental genomes and keep them separated throughout the first mitotic division (Reichmann et al., 2018b). These two bipolar spindles first assemble and congress the parental chromosome sets independently in pro-metaphase. Then, in metaphase, they align their pole-to-pole axes in order to segregate the two chromosome sets in parallel during anaphase. However, if the alignment of the two spindles is perturbed, the parental genomes can be segregated in different directions, leading to gross mitotic aberrations (e.g., formation of binucleated blastomeres or direct cleavage to three or four daughter cells), reminiscent of clinical phenotypes observed in human IVF embryos (Reichmann et al., 2018b).

Unlike most mammalian species, rodent zygotes do not contain centrosomes, with the sperm centrioles appearing to degenerate completely during spermiogenesis (Manandhar et al., 1998; Woolley and Fawcett, 1973). Instead, numerous acentriolar cytoplasmic microtubule organizing centers (MTOCs) are present during the first divisions, and the assembly of the bipolar spindles relies on microtubule self-organization and MTOC clustering (Courtois et al., 2012; Reichmann et al., 2018b). By contrast, nonrodent mammalian zygotes, such as human or bovine, inherit the centrioles from the sperm (Sathananthan et al., 1996; Fishman et al., 2018; Amargant et al., 2021 *Preprint*). Thus, in principle, they have two centers of cytoplasmic microtubule nucleation from the onset of mitosis. However, it is not clear whether these centrioles are in fact fully functional and how spindle assembly proceeds in these species. It might proceed analogously to that in somatic cells, where two centrosomes are the dominant centers of microtubule nucleation and ensure formation of a single bipolar array early in mitosis. Alternatively, the mechanism may be similar as in the mouse zygote involving the self-assembly of two separate bipolar arrays through centrosome-independent pathways. While, on the one hand, human IVF phenotypes would suggest that the mechanism in nonrodents might be similar to that seen in the mouse, on the other hand, the sperm centrioles have generally been assumed to be active (Fishman et al., 2018), which would argue for a single zygotic spindle. For obvious ethical and legal reasons, it was not possible for us to carry out high-resolution real-time imaging of spindle assembly using fluorescent markers in human embryos. We therefore decided to use in vitro–generated bovine zygotes as a nonrodent mammalian model organism to study how zygotic spindle assembly proceeds in the presence of paternal centrioles. This model system resembles the human zygote in several aspects: In addition to inheriting the centrioles paternally, bovine zygotes have a large size (mature human and bovine oocytes have a diameter of 120 µm; Griffin et al., 2006; Otoi et al., 1997), and especially in vitro produced, but also in vivo–conceived preimplantation cattle embryos show a high incidence of postzygotic aneuploidies (Tšuiko et al., 2017). To study zygotic spindle assembly, we combined systematic immunofluorescence (IF) of bovine zygotes, fixed at different stages of the cell cycle, with real-time imaging of live zygotes by light-sheet microscopy during the first mitotic division. Our data clearly indicate that dual spindle assembly is a conserved mechanism, even when paternally inherited centrosomes are present.

## Results and discussion

### Two separate zygotic spindles assemble in the presence of centrosomes

**Figure 1.**
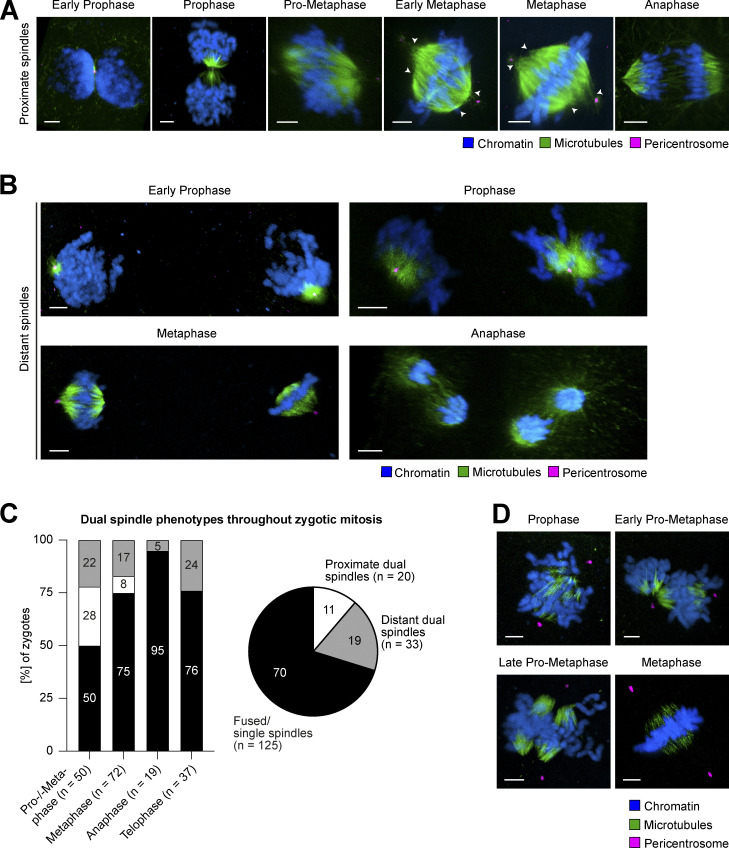
**Dual spindle phenotypes in bovine zygotes. (A and B)** IF of bovine zygotes fixed at 27.5 h after IVF at consecutive stages of mitosis. Maximum intensity projections orthogonal to the estimated spindle axis of confocal sections showing proximate (A) or distant (B) dual spindles. Shown are microtubules (α-tubulin, green), pericentrosomes (Cep192 or Nedd1, magenta), and chromatin (Hoechst, blue). Scale bars, 5 µm. White arrowheads indicate distinct pole clustering for proximate dual spindles. **(C)** Bar graph shows abundance (%) of dual spindle types at different mitotic stages. Pie chart summarizes abundance (%) of dual spindle types throughout mitosis. **(D)** IF staining of bovine zygotes as in A, but following a cold shock on ice for 3 min before fixation. Maximum intensity projections of confocal sections orthogonal to the estimated spindle axis showing that centrosomal microtubules have been depolymerized below the detection limit. Shown are microtubules (α-tubulin, green), pericentrosomes (Nedd1, magenta), and chromatin (Hoechst, blue). Scale bars, 5 µm.

To investigate whether two spindles can form in a mammalian zygote, which inherited two centrioles paternally at fertilization, we analyzed spindle assembly following IVF of bovine oocytes. First, we performed 3D confocal microscopy of zygotes fixed at different stages of the first embryonic mitosis and stained for pericentrosomal material, microtubules, and DNA. In most zygotes, the parental PNi were positioned adjacent to each other in prophase, and in pro-metaphase, we observed that two microtubule arrays formed next to each other around the two parental genomes (proximate spindles; Fig. 1 A). In a small fraction of zygotes, two spindles could still clearly be distinguished in metaphase (8%, *n* = 6/72; Fig. 1 C), by their different lengths, offset between their metaphase plates, and/or distinctly clustered poles (see arrowheads at proximate spindles; Fig. 1 A). In most metaphase zygotes, however, we could not differentiate between dual proximate or single spindles anymore (75%, *n* = 54/72; Fig. 1 C) suggesting that, similar to mouse zygotes, the two adjacent spindles align their longitudinal axes during pro-metaphase and thus appear fused at metaphase stage. In line with this transient nature of dual spindle formation when PNi are close, it was also not possible to clearly distinguish between fused dual spindles and a single spindle at later mitotic stages (Fig. 1 C).

By contrast, in 22% of zygotes in prophase and pro-metaphase (*n* = 11/50), the PNi were further apart and two spindles assembled at a large distance of ∼30–65 µm (distant spindles; Fig. 1, B and C). Such distant dual spindles were evident across all mitotic stages (19% of all zygotes; Fig. 1 C), even including anaphase, and were thus functional for segregating chromosomes.

**Figure S1.**
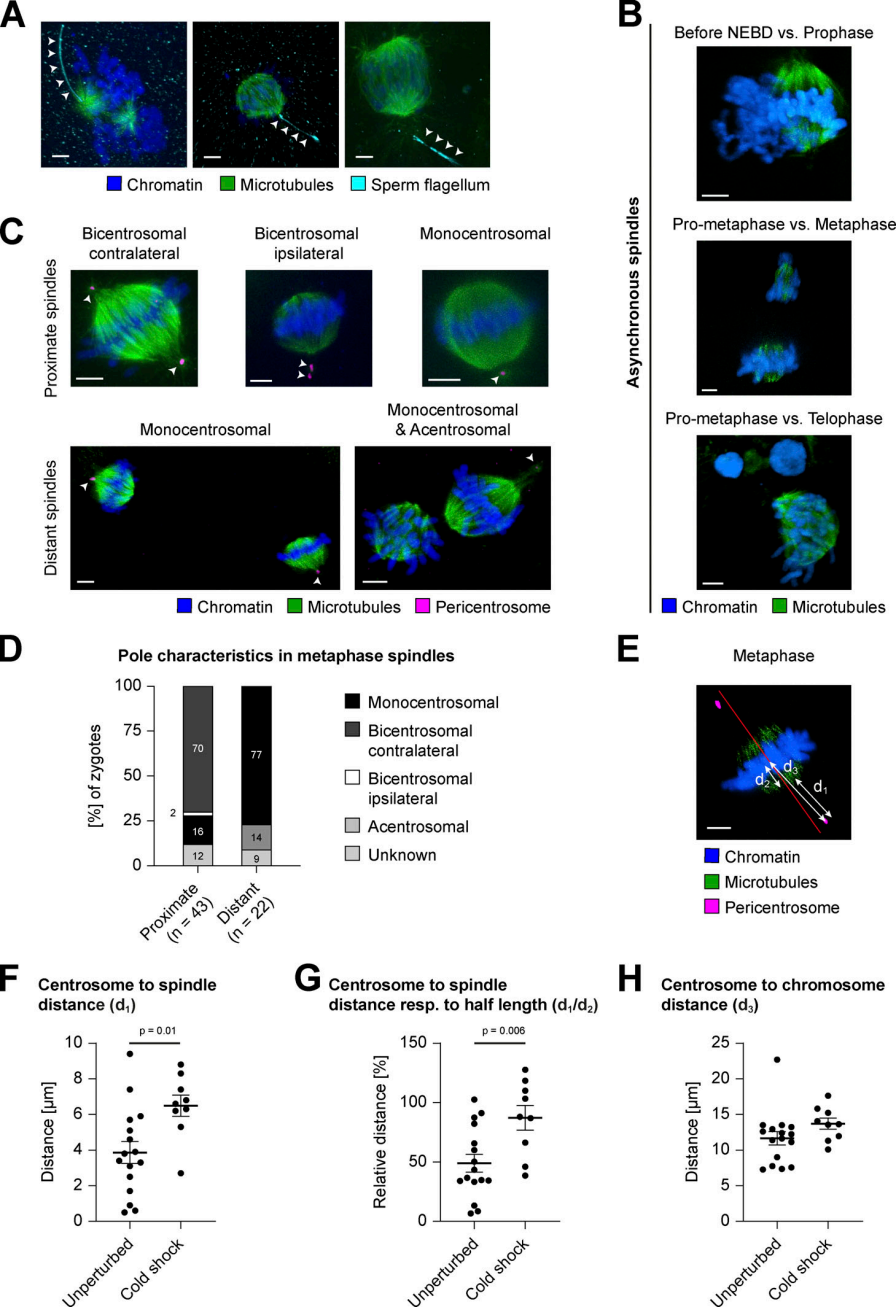
**Dual spindle characteristics in bovine zygotes. (A–C and E)** IF staining of bovine zygotes fixed at 27.5 h after IVF. Maximum intensity projections orthogonal to the spindle axis of confocal sections are shown for microtubules (α-tubulin, green), chromatin (Hoechst, blue), sperm flagellum (acetyl tubulin, cyan), and pericentrosome (Nedd1 or Cep192, magenta). Scale bars, 5 µm. **(A)** Arrowheads indicate spermatozoan flagellum adjacent to one spindle pole, confirming monospermic fertilization. For further details, see Materials and methods. **(B)** Distant dual spindles with distinct mitotic timing inside same cytoplasm. **(C and D)** Diverse centrosome positioning in proximate (fused) and distant dual spindles. **(C)** Arrowheads indicate the number and position of pericentrosomes. **(D)** Abundance (%) of centrosome positions/pole characteristics as illustrated in C. **(E–H)** Comparison of centrosome positioning in bicentrosomal (contralateral) metaphase spindles after IF of unperturbed (*n* = 16) or cold-treated (3 min cold shock on ice, *n* = 9) zygotes. d_1_, centrosome to spindle distance; d_2_, spindle half-length; d_3_, centrosome to chromosome distance. **(E)** Illustration of the measurements in exemplary metaphase spindle after cold treatment (see also Fig. 1 D); red line illustrates projected spindle axis orthogonal to chromosomes. **(F)** Distance in micrometers between centrosomes and spindle body (d_1_, see arrow in E). For assessment of spindle body, see Materials and methods. Error bars indicate SEM distance of unperturbed (3.9 µm) versus cold shock–treated (6.5 µm) zygotes (P = 0.01, significant). **(G)** Relative distance (%) between centrosome and spindle microtubules respective to spindle half-length (d_1_/d_2_, see arrows in E). Error bars indicate SEM distance in unperturbed (49.1%) versus cold shock–treated (87.3%) zygotes (P = 0.006, significant). **(H)** Distance in micrometers between centrosomes and the chromosome centroid (d_3_, see arrow in E). Error bars indicate SEM distance in unperturbed (11.7 µm) versus cold shock–treated (13.7 µm) zygotes (P = 0.15). **(F–H)** Average measurements for both centrosomes from same zygote are depicted. Statistical tests: two-tailed unpaired Student’s *t* test.

Often, the timing of mitotic progression was asynchronous between the two parental PNi. This was especially evident at NEBD, and, albeit more rarely, was also observed in later mitotic stages (Fig. S1 B). The asynchrony suggests that the two PNi cannot only set up two distinct spindles but can also independently regulate their cell cycle progression, even though they share a common cytoplasm. Overall, we could clearly score two spindles around the parental genomes in 30% of all mitotic zygotes we analyzed (*n* = 53/178; Fig. 1 C). This finding is in agreement with a recent paper from Brooks and colleagues, who observed that in 19 of the 49 bovine zygotes (38%) undergoing the first mitotic division, the two parental genomes failed to merge and thus segregated independently (Brooks et al., 2020 *Preprint*).

**Figure S2.**
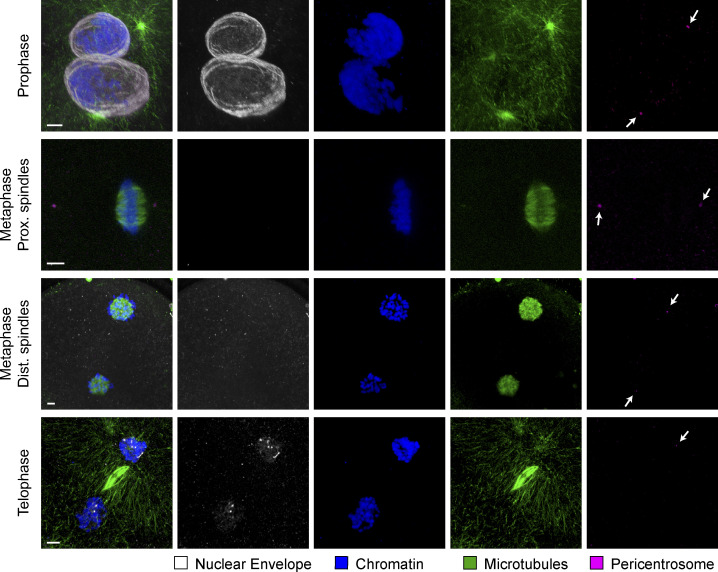
**Localization of nuclear lamina during first mitotic division in the bovine zygote.** IF staining of bovine zygotes fixed at 27.5 h after IVF showing NE localization in consecutive stages of mitosis, from prophase to telophase and in proximate (Prox.) and distant (Dist.) dual spindles. Maximum intensity projections of confocal sections of the spindle volumes are shown for microtubules (α-tubulin, green), pericentrosomes (Nedd1, magenta), chromatin (Hoechst, blue), and NE (lamin B2, gray). Scale bars, 5 µm. Arrows indicate pericentrosome positions.

**Figure S3.**
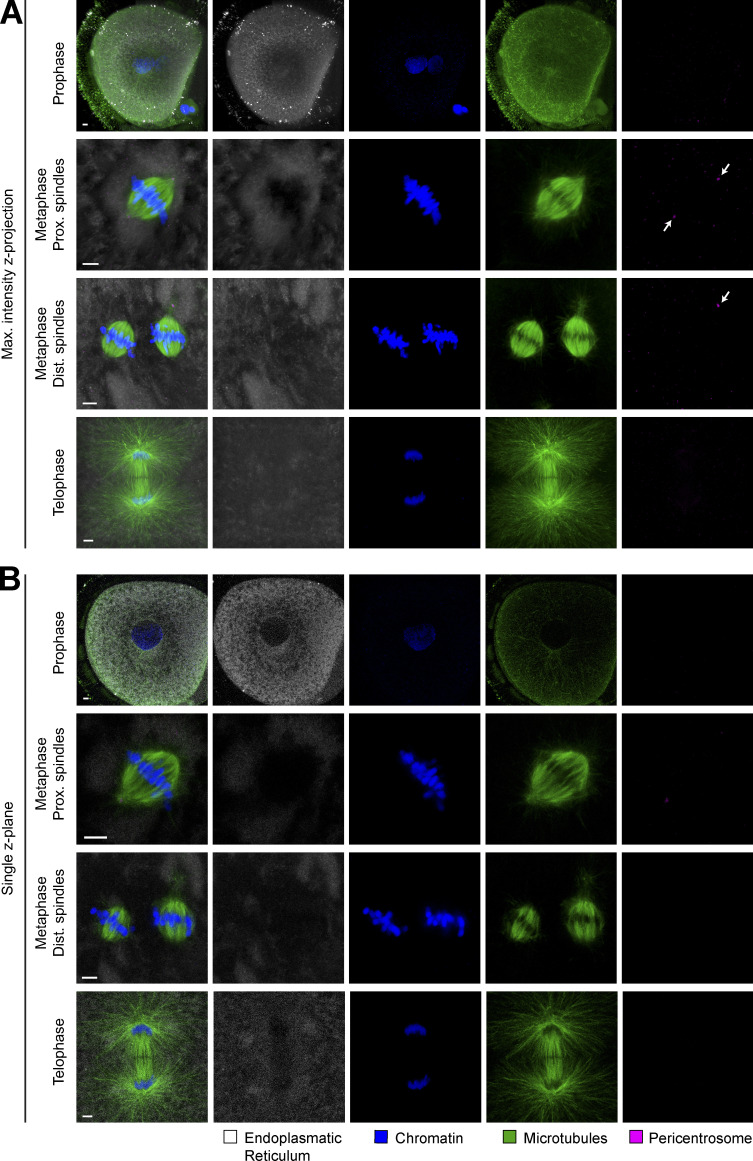
**Localization of endoplasmic reticulum during first mitotic division in the bovine zygote. (A and B)** IF staining of bovine zygotes fixed at 27.5 h after IVF showing ER localization in consecutive stages of mitosis, from prophase to telophase and in proximate (Prox.) and distant (Dist.) dual spindles. **(A)** Maximum intensity projections of confocal sections of entire spindle volumes are shown. **(B)** Single z-planes through the spindles show that no ER barrier could be detected at the spindle intersection. Shown are microtubules (α-tubulin, green), pericentrosomes (Nedd1, magenta), chromatin (Hoechst, blue), and ER (calnexin, cyan). Scale bars, 5 µm. Arrows indicate pericentrosome positions.

In *Caenorhabditis elegans* embryos, where nuclear lamin cannot be phosphorylated and degraded, a physical barrier remains between the two genomes throughout mitosis, which provokes the separation of the parental chromosomes into distinct nuclei within the daughter blastomeres (Velez-Aguilera et al., 2020). To determine whether such remnants of NE or ER might be involved in the spatial separation of parental genomes in bovine zygotes, we performed IF against respective organelle markers lamin B2 and calnexin (Figs. S2 and S3). However, neither for the NE (Fig. S2), nor for the ER (Fig. S3) could we detect any remnants between the parental chromosome masses from NEBD until late anaphase. Since we could clearly observe the first steps of NE reassembly in telophase (Fig. S2, lower panel), we should have detected even small remnants with this approach during earlier stages of mitosis. These data therefore indicate that NEBD is complete in bovine zygotes and that NE remnants do not appear to contribute to spatial separation of parental genomes or dual spindle formation. The diffuse distribution of calnexin signal in bovine zygotes closely resembles previously published ER labeling data in oocytes and zygotes of other mammalian species, including human (FitzHarris et al., 2007; Payne and Schatten, 2003; Audouard et al., 2011; Czajkowska et al., 2020; Balakier et al., 2002; Hölzenspies et al., 2009). Our data demonstrate that ER does not form a physical barrier within the metaphase spindle between proximate parental genomes (maximum intensity projections across whole spindle volumes, Fig. S3 A; single confocal sections through spindle/s or pronucleus [PN], Fig. S3 B).

Together, these results demonstrate that dual spindle assembly (i.e., one around each of the two PNi) also occurs in mammalian zygotes that contain two centrosomes and, if the two PNi are distant, remains pronounced until chromosomes segregate. Since neither NE remnants nor the ER physically separate the two genomes and spindles, it is likely that it is the assembly of the dual spindles that keeps the genomes compartmentalized throughout the whole first mitotic division in bovine zygotes (Cavazza et al., 2021), similarly as in the mouse (Reichmann et al., 2018b).

### Centrosomes distribute variably between the four poles of dual zygotic spindles

Among all metaphase zygotes, 17% presented distant yet bipolar dual spindles (Fig. 1 C). Surprisingly, in most of these spindle pairs, pericentrosomal staining indicated that only one pole of each spindle was associated with a centrosome (monocentrosomal spindles, 77%; Fig. S1, C and D), whereas only few distant dual spindles were acentrosomal (14%; Fig. S1, C and D) or could not be scored due to poor pericentrosomal staining (9%; Fig. S1 D). By comparison, proximate, closely aligned (or fused) spindles in metaphase mostly showed one centrosome at each of the two spindle poles (bicentrosomal contralateral spindles, 70%; Fig. S1, C and D), although we also observed monocentrosomal spindles (16%; Fig. S1, C and D) and, in one case, a spindle with both centrosomes at the same pole (bicentrosomal ipsilateral spindle, 2%; Fig. S1, C and D). To summarize, most commonly both centrosomes localized to the opposite poles in proximate/fused spindles, and in distant spindles each spindle showed one polar centrosome. Nonetheless, centrosome distribution varied, and we observed bipolar microtubule arrays that were able to segregate the chromosomes even if one or both poles lacked a centrosome. This suggests that the presence of centrosomes is not essential for spindle assembly and chromosome segregation in bovine zygotes. Whether distant monocentrosomal spindles are a consequence of incomplete pronuclear migration or abnormal PNi–centrosome interaction remains to be determined.

### Centrosomes are only weakly linked to the spindle body

In both mono- and bicentrosomal spindles, the centrosomal microtubules appeared sparse and connected the centrosome to the body of the spindle only weakly. This was especially evident in fully assembled spindles from early metaphase onwards (Fig. 1, A and B) and is in contrast to somatic spindles in many eukaryotic cell types, especially the typical mitotic model systems of marsupial and hamster cells, where stable kinetochore fibers connect the pericentrosome directly to the chromosomes (McDonald et al., 1992; Brinkley and Cartwright, 1971). To examine the strength of the connection between the centrosomal asters and the spindle body in the zygote, we subjected zygotes to a brief cold treatment to depolymerize unstable microtubules before fixation. Under these conditions, the microtubule bundles in the spindle body around the chromosomes were preserved, but the microtubules emanating from the centrosomes decreased to below the detection limit at all mitotic stages (Fig. 1 D). After removing unstable microtubules in this manner, the gap between the spindle body and the centrosome increased significantly from 3.9 to 6.5 µm on average in metaphase (d_1_, P = 0.01; Fig. S1, E and F), a distance that is similar to the spindle half-length after cold treatment (d_2_; Fig. S1 E). Thus, the ratio between the centrosome-to-spindle distance and the spindle half-length increased from ∼49% in unperturbed zygotes to 87% at cold treatment (d_1_/d_2_, P = 0.006; Fig. S1 G). In addition, the distance between centrosomes and the metaphase plate increased after cold treatment, although this was not statistically different (d_3_, P = 0.15; Fig. S1, E and H). Additionally, we noted that in cold-treated zygotes, the two separate spindles forming around the parental genomes became more clearly visible, because a large gap had opened between the remaining stable microtubule arrays as a result of the cold treatment (late pro-metaphase; Fig. 1 D). These data demonstrate that the sparse microtubules connecting the centrosome to the spindle body as well as the microtubules between the dual spindles are unstable. This suggests that the centrosomes are only weakly linked to the spindle body and that the connection between the two spindles is also driven by dynamic microtubules.

### Centrosomes do not make a major contribution to metaphase spindle architecture

**Figure 2.**
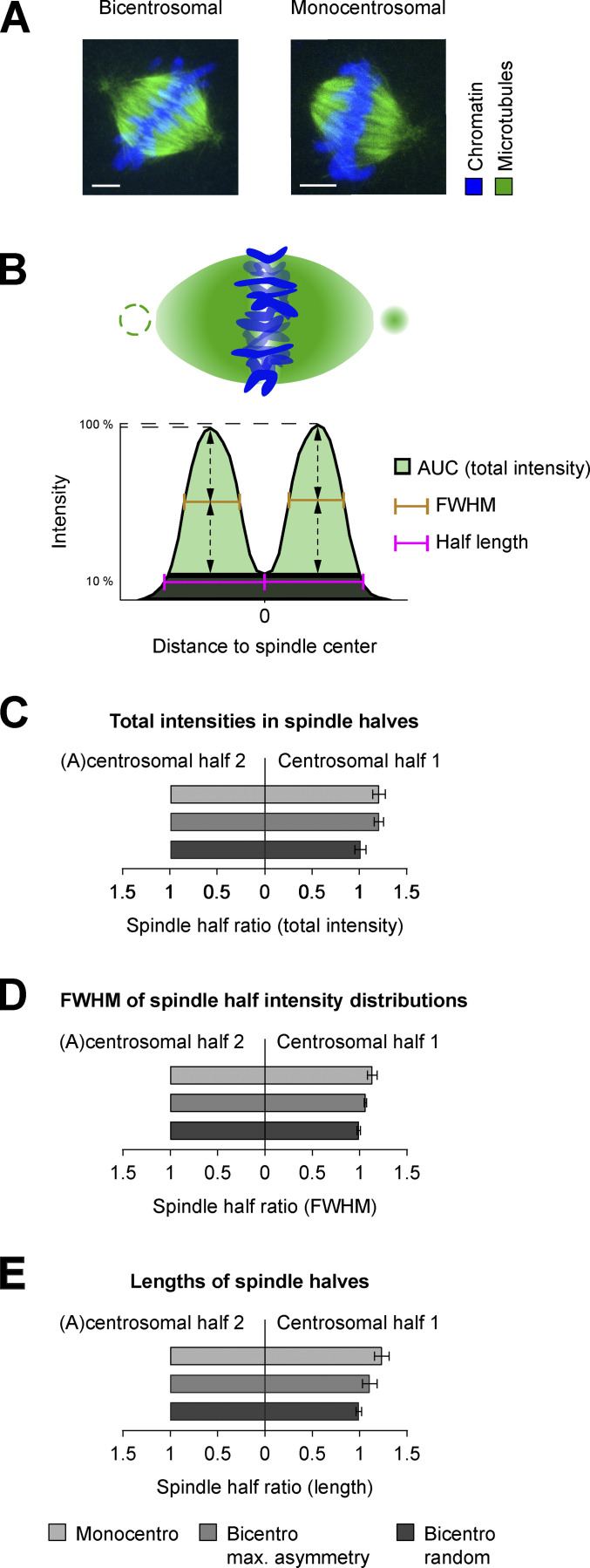
**Quantitative comparison of proximate and distant dual spindles. (A)** Exemplary IF data subjected for quantitative comparison of proximate bicentrosomal and distant monocentrosomal spindles (see also Fig. 1, A and B). Metaphase spindles of bovine zygotes fixed at 27.5 h after IVF. Maximum intensity projections over the imaging plane (z) are shown. **(B)** Schematic representation of zygotic metaphase spindle and total intensity distribution along determined spindle axis orthogonal to the chromosomes in bicentrosomal contralateral and monocentrosomal spindles. Note dashed circle to illustrate second centrosome in bicentrosomal spindles and missing centrosome in monocentrosomal spindles. FWHM was considered as an estimate of intensity distribution and AUC as a sum of total intensities in each spindle half; the half-lengths were calculated as distances between the intensity distribution’s valley (0 position) and the most distant positions along the axis, where the total intensity was 10% of the respective maximum. **(C–E)** Ratiometric comparison of total intensity (C), FWHM (D), and of the length (E) between the halves of the spindle types. For distant monocentrosomal spindles (Monocentro; *n* = 11 from 6 embryos), absolute measurements were normalized to acentrosomal half. For proximate bicentrosomal contralateral spindles (Bicentro max. asymmetry; *n* = 16), absolute measurements were normalized to the spindle half with lower sum intensity or shorter FWHM and length. Randomly normalizing the halves of these bicentrosomal spindles (Bicentro random; *n* = 16) shows that such small asymmetries are usually averaged out upon random comparison. Error bars indicate SEM. Statistical tests: two-tailed unpaired Student’s *t* test. **(C)** Mean intensity ratio between centrosomal and acentrosomal halves of monocentrosomal spindles (Monocentro, 1.21) was comparable to the ratio between brighter and dimmer halves in bicentrosomal spindles (Bicentro max. asymmetry, 1.21, P = 0.99). **(D)** Comparable mean ratio of FWHM of monocentrosomal spindle halves (Monocentro, 1.14) and bicentrosomal spindle halves when normalized to highlight natural asymmetry (Bicentro max. asymmetry, 1.06, P = 0.12). **(E)** Overall similar mean ratio of spindle half-lengths in mono- (Monocentro, 1.24) and bicentrosomal (Bicentro max. asymmetry, 1.11) spindles (P = 0.07) when normalized to highlight natural asymmetry.

**Figure S4.**
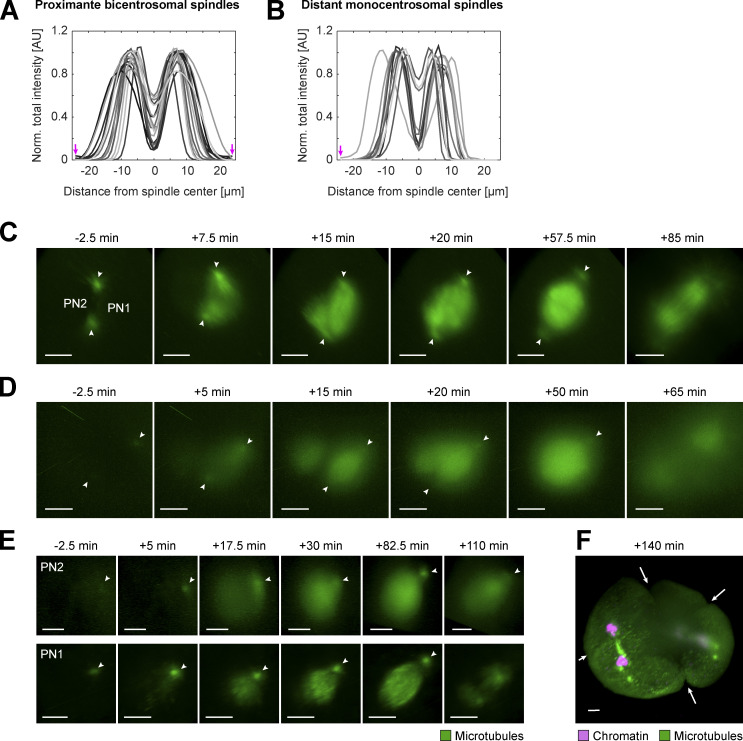
**Comparing microtubule distribution in proximate bicentrosomal and distant monocentrosomal spindles in fixed and live bovine zygotes. (A and B) **Intensity distribution of α-tubulin IF in 2D sections along the calculated spindle axis orthogonal to the metaphase chromosomes in both proximate bicentrosomal contralateral (A, *n* = 16) and distant monocentrosomal (B, *n* = 11 from six embryos) spindles (see also Fig. 2, A and B). Arrows indicate positions of centrosomes. **(C–E)** Respective 3D-rendered images of fluorescence from microtubule markers (EGFP-MAP4 and EB3-mEGFP2) in the pronuclear volumes of zygotes shown in Fig. 3, A–C to highlight dual spindles, and centrosome positions (arrowheads). Timings respective to synchronous pro-NEBD (NEBD) or NEBD of the first PN (PN1) in case of asynchrony. PN2, lagging PN. Projected scale bars, 10 µm.** (F) **3D-rendered image of fluorescence from microtubule marker (EB3-mEGFP2, green) and chromatin marker (H2B-mCherry, magenta) of zygotic volume (same zygote as Fig. 3 C and Fig. S2, D and E) after background correction (median-based denoising). Arrows indicate multiple ingression sites at 140 min after NEBD as a consequence of distant dual spindles.

We next asked whether the weakly connected polar centrosomes influenced zygotic spindle architecture significantly. To answer this, we took advantage of the frequent occurrence of a monocentrosomal spindle configuration in zygotes showing distant dual spindles (Fig. S1, C and D). Although these separate spindles were smaller than proximate spindles, because they contained only one parental genome, they naturally offered the possibility to investigate whether the presence of a centrosome at only one pole induces a strong architectural asymmetry between the spindle halves. To determine the general base line of asymmetry between spindle halves in bovine zygotes, we also calculated the structural differences between the halves of proximate fused spindles that had a centrosome at both poles (e.g., bicentrosomal contralateral; Fig. S1 C). To measure symmetry, we computationally segmented the tubulin signal and quantified its spatial intensity distribution along the axis of the spindle orthogonal to the metaphase plate (Fig. 2, A and B; and Fig. S4, A and B; for a detailed description, see Materials and methods). To compare the microtubule mass on both sides of the spindle, the total tubulin intensity within each spindle half, which corresponds to the area under the intensity distribution curve (AUC) of each half, was calculated and plotted ratiometrically (Fig. 2, B and C). For monocentrosomal spindles, the total tubulin intensity in the centrosomal half of the spindle was slightly higher than that in the acentrosomal half (mean ratio = 1.2; Fig. 2 C, Monocentro). This slight asymmetry was very similar to that between bicentrosomal spindle halves, when comparing the brighter to the dimmer half (mean ratio = 1.2; Fig. 2 C, Bicentro max. asymmetry). This indicates that a polar centrosome does not increase the microtubule mass in the associated spindle half by more than 20%, which is indistinguishable from the normal variation in microtubule mass between the halves of a bicentrosomal spindle (P = 0.99). When just randomly comparing the intensities between the two halves in bicentrosomal spindles, these naturally occurring asymmetries are averaged out (mean ratio = 1; Fig. 2 C, Bicentro random).

Even though the presence of a centrosome does not significantly change the amount of tubulin, it could still broaden its spatial distribution along the spindle axis away from the chromosomes/equator. To investigate such subtle changes in spindle architecture, we measured the full width at half maximum (FWHM) of the intensity distribution on each side of the metaphase plate for mono- and bicentrosomal spindles and again compared them ratiometrically (Fig. 2, B and D). In monocentrosomal spindle halves, the FWHM increased slightly on the centrosomal side (mean ratio = 1.1; Fig. 2 D, Monocentro), but the difference was not significantly different from that in bicentrosomal spindles when comparing brighter with the dim halves (mean ratio = 1.1; P = 0.12; Fig. 2 D, Bicentro max. asymmetry). This indicates that a polar centrosome does not broaden the extension of dense tubulin away from the metaphase plate by more than 10%, indistinguishable from the normal variation found in bicentrosomal spindles. To investigate whether a centrosome might increase spindle length, for example by stabilizing microtubule bundles, we also compared the lengths of the halves within mono- and bicentrosomal spindles (Fig. 2, B and E). In monocentrosomal spindles, we observed few asymmetric spindles, but on average, the centrosomal half was only slightly longer than the acentrosomal half (mean ratio = 1.2; Fig. 2 E, Monocentro). This difference was not significantly different from the length asymmetry observed in bicentrosomal spindles (mean ratio = 1.1, P = 0.07; Fig. 2 E, Bicentro max. asymmetry). In summary, our quantitative analysis of the two halves of mono- or bicentrosomal spindles demonstrates that the presence of a centrosome at one pole of a zygotic metaphase spindle does not introduce a significant bias in spindle structure beyond the naturally occurring asymmetry, which can be observed in normal bicentrosomal spindles. For the structural parameters of total microtubule mass, the spatial distribution of microtubule mass, and the spindle half-length, the differences were within the range of naturally occurring variability in bicentrosomal spindles, which is easily averaged out when randomly comparing the two halves (Fig. 2, C–E, Bicentro random). Combined with the finding that centrosomes are only weakly linked to the spindle body and that mono- and acentrosomal spindles could segregate chromosomes, this overall high structural symmetry of monocentrosomal spindles provides additional evidence that suggests that polar centrosomes only have a weak influence on the assembly of the mitotic spindle in bovine zygotes.

**Figure S5.**
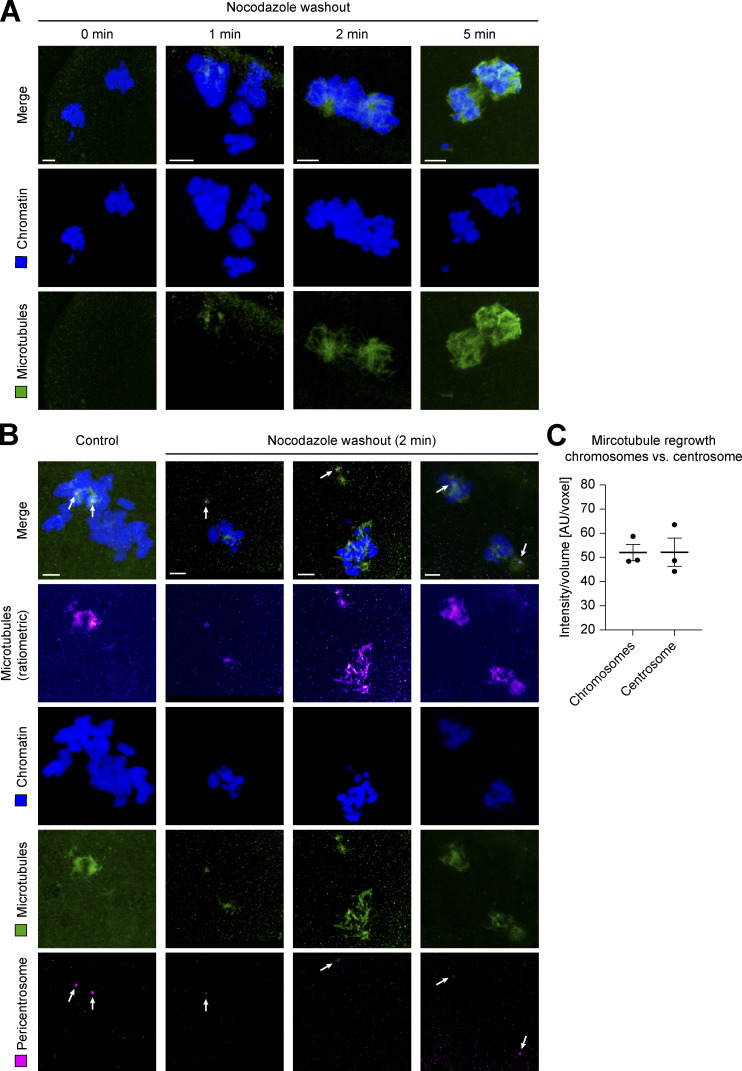
**Microtubule regrowth at chromosomes and centrosomes after nocodazole washout during pro-metaphase of first mitotic division in the bovine zygote.** **(A)** IF staining of bovine zygotes fixed after 0, 1, 2, and 5 min of microtubule regrowth after washout from incubation at 5 µM nocodazole for 4 h (starting at 27.5 h after IVF). Maximum intensity projections of confocal sections are shown for chromatin (Hoechst, blue) and microtubules (α-tubulin, green). Scale bars, 5 µm. **(B)** IF staining of bovine zygotes fixed at 27.5 h after IVF (control) and of three example zygotes after 2 min of nocodazole washout as in A. Maximum intensity projections of confocal sections are shown for chromatin (Hoechst, blue), microtubules (α-tubulin, green), and pericentrosome (Nedd1, magenta). Additional ratiometric representation of microtubules (α-tubulin, fire). Arrows indicate pericentrosome positions. Scale bars, 5 µm. **(C)** Quantification of microtubule mass (intensity/volume) at chromosomes and the centrosome after nocodazole washout for 2 min (*n* = 3). Equal growth at both sites shown by equal intensities at both sites (mean intensity of 50.0 at chromosomes vs. 50.1 at centrosomes). Error bars indicate SEM intensity.

To directly compare the strength of microtubule nucleation by centrosomes and chromosomes at the beginning of spindle assembly, we also performed a microtubule regrowth assay after completely depolymerizing them with nocodazole in pro-metaphase (Fig. S5 A). In this assay, centrosomes and chromosomes are often spatially well separated and, since the NE is already broken down, can start nucleation simultaneously after removal of the inhibitor in the common mitotic cytoplasm. Within only 2 min after washing out nocodazole, microtubules regrew equally strong around chromosomes as compared with centrosomes (ratio between microtubule signal at chromosomes and centrosomes ∼1; Fig. S5, B and C).

Both the surprising symmetry of acentrosomal and centrosomal spindle halves and strong microtubule nucleation around chromosomes after nocodazole washout suggest that centrosome-independent pathways play a major role for zygotic spindle assembly and maintenance. Nevertheless, centrosomes may have other important functions in the zygote, such as pronuclear migration and the recently reported chromosome clustering at the pronuclear interface (Cavazza et al., 2021).

### Real-time imaging reveals the dynamic process of dual spindle assembly

Although we analyzed many zygotes (1,421, of which 178 were undergoing mitosis), it was difficult to infer the precise order of the dynamic steps of dual spindle assembly in the presence of paternal centrosomes from snapshots of individually fixed embryos, primarily because of poor synchronicity and variability in pronuclear position. We therefore decided to visualize spindle assembly in real time in live bovine zygotes. We adapted our micromanipulation and imaging pipelines, which we developed for in toto imaging of preimplantation mouse embryos (Strnad et al., 2016; Reichmann et al., 2018a), for the larger and more strongly scattering bovine embryos (∼120 µm in diameter, nearly twice as big as the ∼70 µm mouse embryos; Griffin et al., 2006; Otoi et al., 1997; for details, see Materials and methods). Using mRNA microinjection at the pronuclear stage (Jaffe and Terasaki, 2004), we transiently expressed live fluorescent markers for chromosomes (histone 2B [H2B]) and the growing tips or lattice of microtubules (end-binding protein 3 [EB3] or microtubule-associated protein 4 [MAP4]). The inverted and low-dose light-sheet microscope allowed us to maintain IVF culture conditions for bovine embryos and image them in 3D with a high temporal resolution of 2.5 min throughout the first division. These novel real-time datasets of bovine zygotic mitosis clearly demonstrated that, indeed, two microtubule arrays assembled around the parental genomes in the presence of two centrosomes. Live imaging of a total of 21 dividing embryos revealed several different modes by which the two assembling spindles incorporated the two centrosomes, explaining the generation of the very different centrosome distributions that we had observed in fixed zygotes.

**Figure 3.**
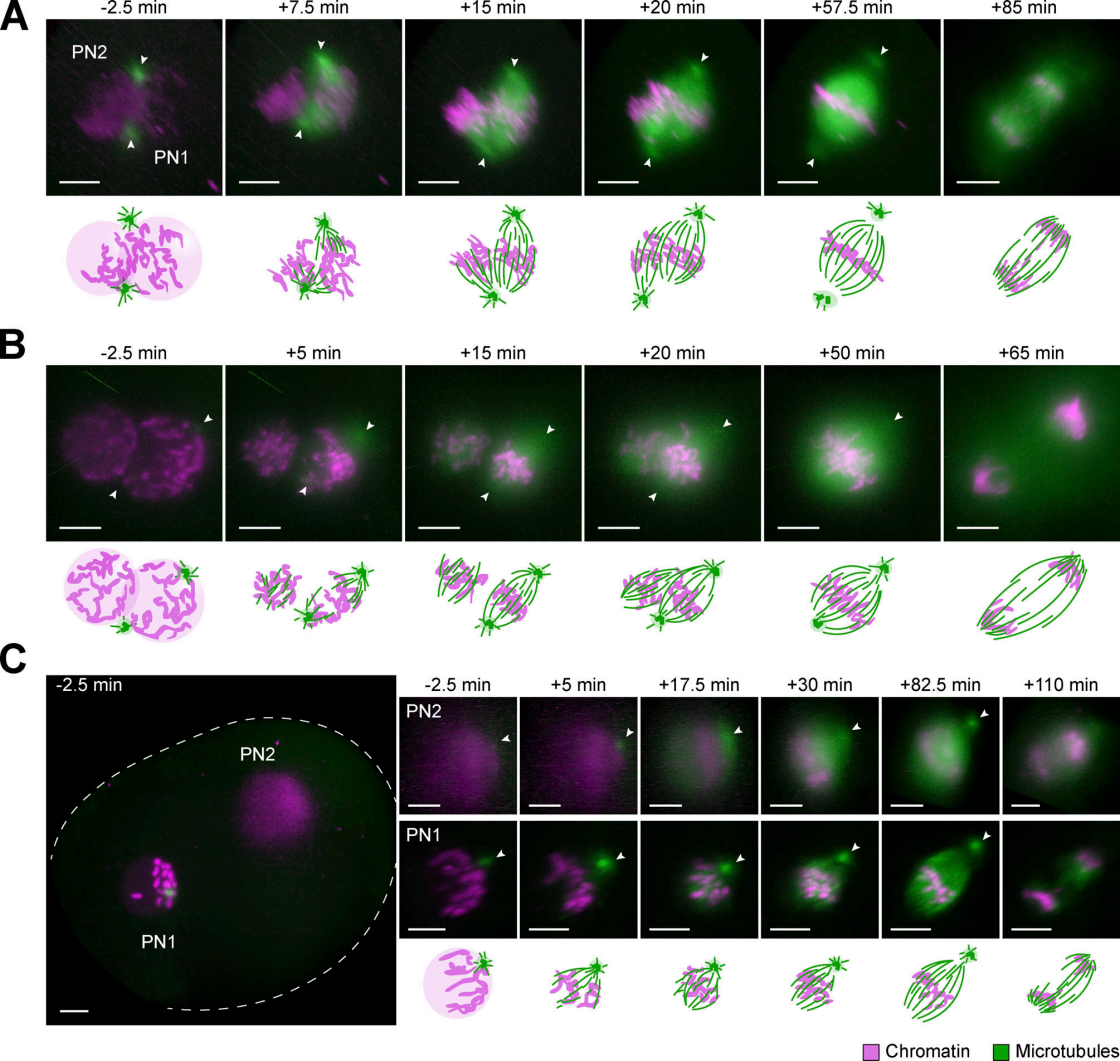
**Assembly and dynamics of proximate and distant dual spindles in live bovine zygotes. (A–C)** Bovine zygotes expressing microtubule markers (EGFP-MAP4 in A or EB3-mEGFP2 in B and C, green) and chromatin marker (H2B-mCherry, magenta) were imaged by light-sheet microscopy every 2.5 min throughout mitosis and for up to 6 h in total. 3D-rendered images of pronuclear volumes are shown. Overview image to illustrate pronuclear distance (C) is a background-corrected (median-based denoising) overlay of maximum intensity projections over z of both pronuclear volumes within the zygote (zygotic rim indicated by dashed lines). Timings are respective to synchronous pro-NEBD (B) or to NEBD of leading PN (PN1) in case of asynchrony (A and C). PN2, lagging PN. Arrowheads indicate positions of centrosomes. Projected scale bars, 10 µm. **(A and B) **Most frequent (A) and most pronounced (B) example of proximate dual spindle assembly. **(C)** Example of distant dual spindle assembly with two individual monocentrosomal spindles throughout mitosis.

Consistent with the observations in fixed embryos, asynchronous NEBD of the two PNi was very common (*n* = 19/21), with a delay between the leading and lagging PN ranging from 2.5 to 7.5 min. Independent of synchronicity, microtubules often accumulated within the original pronuclear volumes, and two small microtubule asters formed around the centrosomes. In most of the zygotes, the parental PNi had come into proximity before NEBD (*n* = 20/21). The centrosomes were also mostly in contact with the pronuclear surfaces (Fig. 3, A and B; and Fig. S4, C and D). How the centrosomal asters were then associating with the two spindles forming around the chromosomes largely depended on their original orientation respective to the PNi. Most commonly (∼60%), both centrosomes were wedged between the two NEs and thus associated with both parental genomes. From here, they were usually incorporated into one pole of each of the two developing spindles in a revealing dynamic process, where both asters initially associated with the spindle that formed around the “leading” PN, undergoing NEBD first (e.g., Figs. 3 A and S4 C, PN1, 7.5 min). Once the second PN also initiated NEBD (Figs. 3 A and S4 C, PN2, 15 min), microtubules transiently accumulated around its chromosomes and a second (half-) spindle formed between one of the centrosomes and the second genome. In cases where the spindle orientation relative to the light-sheet allowed high-resolution imaging of this step, we observed that the microtubules accumulating around the second genome pulled one centrosome away from the first spindle, incorporating it instead into the second, initially often monopolar array (Figs. 3 A and S4 C, 15 and 20 min after NEBD; see Video 1). Subsequently, the second spindle also became bipolar and the two spindles aligned their axes in parallel (see Video 1). Finally, we could recognize the fused dual spindle with an overall round appearance and broad poles that we had often observed in fixed embryos (compare Figs. 3 A and S4 C, 57.5 min with Fig. S1 C, bicentrosomal contralateral). Interestingly, also in live embryos, some of these fused proximal metaphase spindles still had polar centrosomes, which were positioned slightly off center and were only weakly connected to the spindle body (Figs. 3 A and S4 C, 20 and 57.5 min after NEBD; see Video 1). Rarely, no dominant initial bipolar array was developed, but instead, two monopolar and monocentrosomal spindles formed around the two PNi, eventually combining into a bipolar array.

**Figure 4.**
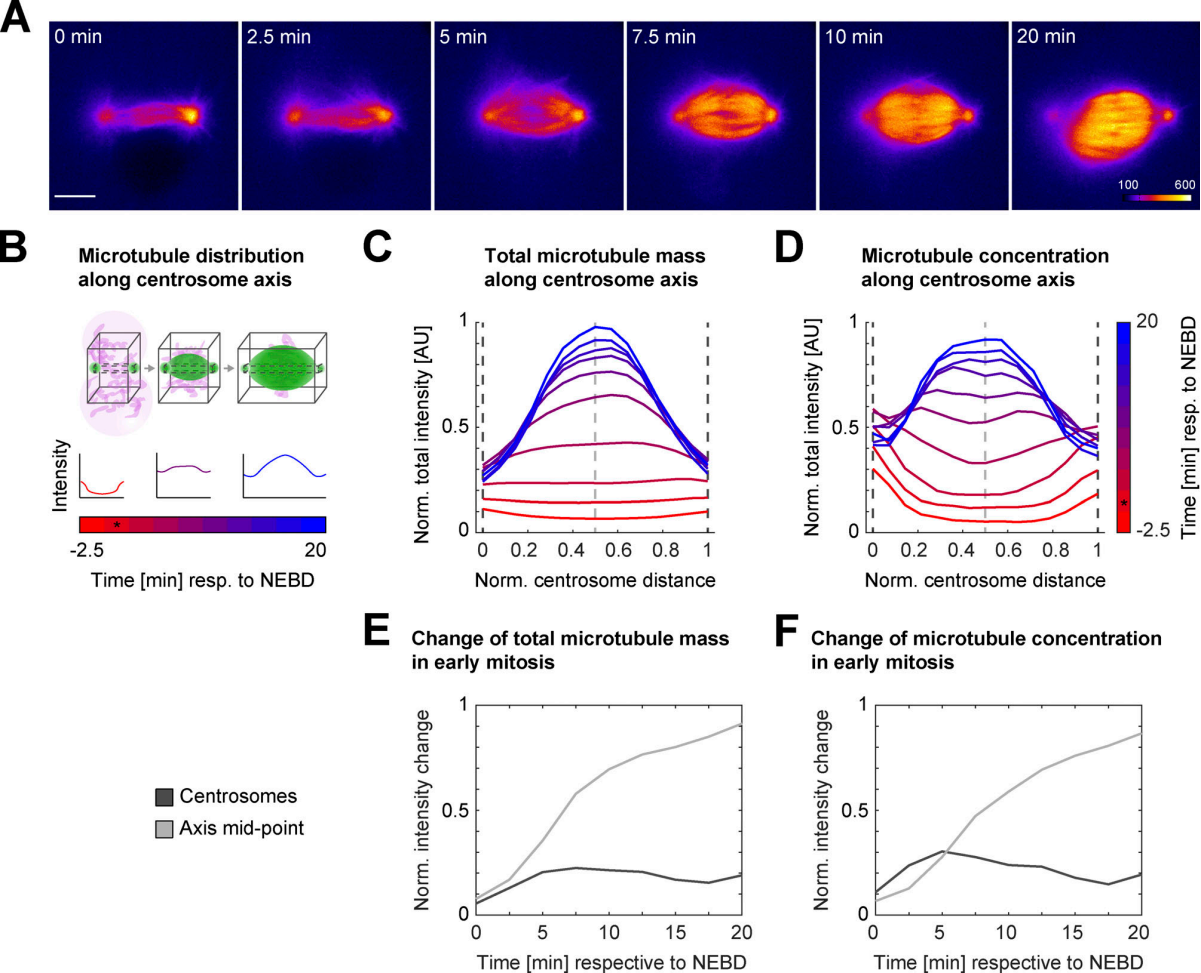
**Analysis of dynamic distribution of spindle microtubules in live bovine zygotes. (A–F)** Microtubule signal (EGFP-MAP4 or EB3-mEGFP2) from live imaging of bovine zygotes by light-sheet microscopy every 2.5 min with a spindle assembly type as described in Fig. 3 A was analyzed for 10 time points starting 2.5 min before NEBD of the leading PN (PN1) or both PNi. **(A)** Pseudocolor representation of EGFP-MAP4 signal within single planes through the centers of intensities at centrosomes. Corresponding lookup table is depicted in last frame of the time series. 6 of 10 analyzed time frames were selected to visualize critical time points for microtubule redistribution in early spindle assembly. Time in minutes respective to NEBD. Scale bar, 10 µm. **(B–D)** Measuring intensity distribution of microtubule signal along the centrosomal axis over time. **(B)** Scheme illustrating the measurements. After background subtraction, total microtubule intensities were calculated for 15 equidistantly distributed 2D slices within the black cuboids along the centrosomal axis for the different time points. NEBD is marked by an asterisk. Black cuboid with solid line encompasses 351 × 351 pixel–sized slices to measure entire microtubule intensity or mass along the spindle axis. Small black cuboid with dashed line encompasses 15 × 15 pixel–sized slices to indicate relative microtubule concentrations. Maximum normalized total intensities along the normalized centrosome distance were annotated. **(C and D)** Average distribution of maximum normalized microtubule intensities along centrosomal axis indicating relative microtubule mass within spindles over time (C; black cuboid with solid line, as described in B) and relative microtubule concentrations (D; black cuboid with dashed line, as described in B); *n* = 6 zygotes. Dashed lines mark the position of the 2D slice through the centrosomes (lines in dark gray) and the centrosome axis midpoint (line in light gray). Color gradient from red to blue indicates time in minutes respective to NEBD. Time of NEBD is indicated by an asterisk. **(E and F)** Average change of normalized total microtubule intensity for total microtubule mass (E) and relative microtubule concentrations (F) over time from NEBD until 20 min after NEBD, at centrosomes and the centrosome axis midpoint, indicated by dashed lines in dark and light gray in C and D, respectively; *n* = 6 zygotes. For intensity change at centrosome, mean intensity of both centrosomes was calculated.

**Video 1.** **Time-lapse imaging of mitotic live bovine zygote expressing EGFP-MAP4 (green) and H2B-mCherry (magenta) after mRNA injection at pronuclear stage.** Time resolution, 2.5 min. Scale bar, 10 µm. Movie shows 30 frames/s. Recording starts at 2.5 min before NEBD of the leading PN (PN1). Movie shows spindle assembly in zygote depicted in Figs. 3 A and S4 C. It is also an example used for analysis (see Fig. 4, C–F).

In one striking example, we could distinguish the separate initial arrays over several minutes (Figs. 3 B and S4 D). Here, the two centrosomes were associated with opposite sides of only one PN and both centrosomes remained associated with the first spindle that formed around this PN. The second genome then clearly nucleated microtubules independently of centrosomes, forming a more spherical bipolar microtubule array. It first increased its microtubule mass before merging with the first, bicentrosomal array, pole by pole (Figs. 3 B and S4 D; 15–50 min after NEBD; see Video 2). Overall, in the embryos showing dual spindle assembly around adjacent PNi with closely associated centrosomes (Fig. 3, A and B), the first mitosis usually resulted in a symmetrical two cell embryo (∼85%).

**Video 2.** **Time-lapse imaging of mitotic live bovine zygote expressing EB3-mEGFP2 (green) and H2B-mCherry (magenta) after mRNA injection at pronuclear stage.** Time resolution; 2.5 min. Scale bar, 10 µm. Movie shows 30 frames/s. Recording starts at 2.5 min before synchronous NEBD. Movie shows spindle assembly in zygote depicted in Figs. 3 B and S4 D.

**Figure S6.**
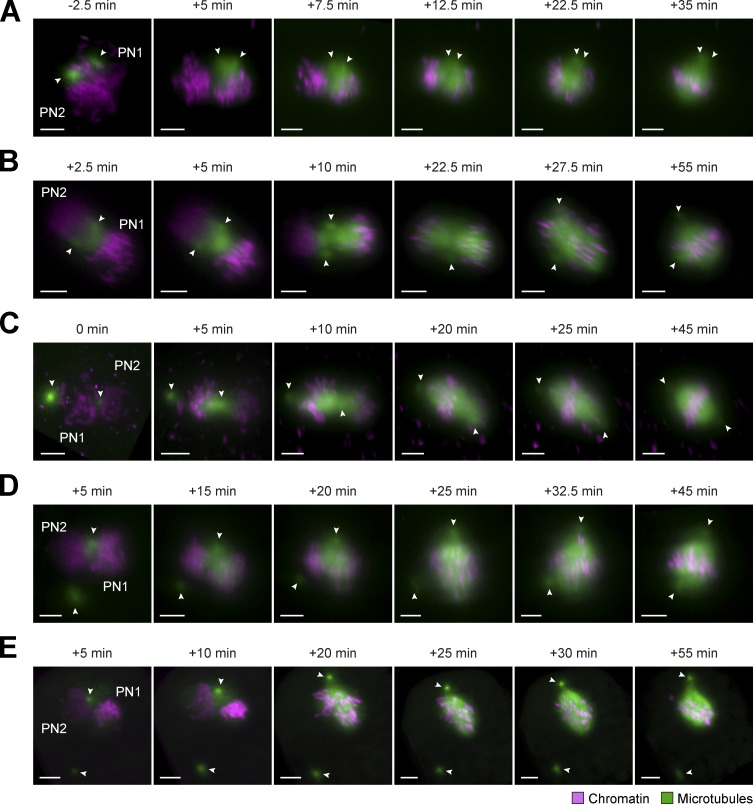
**Miscellaneous spindle assembly modes around proximate parental genomes in live bovine zygotes. (A–E)** Bovine zygotes expressing microtubule markers (EGFP-MAP4 in A–D or EB3-mEGFP2 in E, green) and chromatin marker (H2B-mCherry, magenta) were imaged by light-sheet microscopy every 2.5 min throughout mitosis and for up to 6 h in total. 3D-rendered images of pronuclear volumes after background correction (median-based denoising) show examples of spindle formation and dynamics from prophase to metaphase. Indicated timings respective to synchronous pro-NEBD or to NEBD of first PN (PN1) in case of asynchrony. PN2, lagging PN. Arrowheads indicate positions of centrosomes. Projected scale bars, 10 µm. **(A–C)** Spindle assembly modes around adjacent PNi, where centrosomes localized at PN surfaces, but not at the PN interphase junctions (*n* = 4). Centrosomes either localized in proximity to each other, but not perfectly at PN interphase (A and B) or at opposite sides of same PN, with only one centrosome at PN interphase (C). **(D and E)** Spindle assembly around adjacent PNi, where only one centrosome localized to PN surface/interphase and the second was randomly positioned in cytoplasm without clear nuclear attachment (*n* = 3).

For the remaining zygotes with proximal PNi and associated centrosomes, dual spindle assembly was also evident (Fig. S6, A–C). However, since the centrosomes were originally not located at opposite sites along the pronuclear interphase, the spindle configurations were more variable from embryo to embryo. Instead of assembling a dominant bipolar array, two spherical and/or monopolar spindles assembled around the two genomes seemingly independent of the centrosomes. In the initially forming spindles, centrosome positions ranged from polar but ipsilateral (Fig. S6 A, >7.5 min after NEBD) to apolar (Fig. S6 B, 10–22.5 min after NEBD). As mitosis progressed, the centrosomes were incorporated into different parts of the spindles. However, neither positioning ipsilaterally at one pole (Fig. S6 A, 35 min after NEBD) nor at the spindle midzone (Fig. S6 B, 55 min after NEBD) was corrected.

We also observed spindles with completely dissociated centrosomes (Fig. S6, D and E; *n* = 3/21). Here, only one of the centrosomes was in the proximity of the PNi at NEBD, while the other was far away in the cytoplasm (Fig. S6, D and E, 5 min after NEBD). Again, two microtubule arrays formed that eventually merged into a single bipolar spindle with no centrosome at one pole. If close enough, the second centrosome could be pulled in by the fully formed spindle (Fig. S6 D, 25–45 min after NEBD), but if far away, it remained isolated in the cytoplasm (Fig. S6 E). It would be interesting, in the future, to understand which intrinsic mechanisms could be responsible for the rather frequent phenomenon of centrosome displacement. It is possible that it results from an impaired connection of centrosomes to microtubules and the pro-NE. Such disruptive connection could impair both, centrosome separation and movement.

One of the live-imaged zygotes had its two PNi positioned ∼60 µm apart. Strikingly, its two bipolar microtubule arrays remained separate until chromosome segregation (Figs. 3 C and S5 E; *n* = 1/21). Consistent with most of the fixed embryos with distant dual spindles (Fig. S1, C and D), each PN of this live zygote was associated with one centrosome (Fig. 3 C, 5 min, arrowheads). After NEBD, initially two monocentrosomal spindles formed, remaining over 50 µm apart, which bipolarized and progressed to chromosome segregation (Figs. 3 C and S4 E and Video 3 and Video 4). This configuration did not result in a normal cleavage into a symmetrical two cell embryo but exhibited several mitotic errors, including ingression of multiple cleavage furrows and failure of cytokinesis (Fig. S4 F and Video 5).

**Video 3.** **Time-lapse imaging of mitotic live bovine zygote expressing EGFP-MAP4 (green) and H2B-mCherry (magenta) after mRNA injection at pronuclear stage.** Time resolution, 2.5 min. Scale bar, 10 µm. Movie shows 30 frames/s. Recording starts at 2.5 min before NEBD of the leading PN (PN1). Movie shows spindle assembly around the volume of the lagging PN (PN2) in one zygote, also depicted in Figs. 3 C and Fig. S4, E and F.

**Video 4.** **Time-lapse imaging of mitotic live bovine zygote expressing EGFP-MAP4 (green) and H2B-mCherry (magenta) after mRNA injection at pronuclear stage.** Time resolution, 2.5 min. Scale bar, 10 µm. Movie shows 30 frames/s. Recordings start at 2.5 min before NEBD of the leading PN (PN1). Movies show spindle assembly around the volume of the leading PN (PN1) in one zygote, also depicted in Fig. 3 C and Fig. S4, E and F.

**Video 5.** **Time-lapse imaging of mitotic live bovine zygote expressing EGFP-MAP4 (green) and H2B-mCherry (magenta) after mRNA injection at pronuclear stage. **Time resolution, 2.5 min. Scale bar, 10 µm. Movie shows 30 frames/s. Recordings start at 2.5 min before NEBD of the leading PN (PN1). Movie shows distant dual spindle assembly in the context of the entire imaged volume of one zygote, also depicted in Fig. 3 C and S4, E and F.

Together, our observations in living zygotes were fully consistent with the results obtained by IF and explained the temporal sequence of the spindle assembly intermediates we had observed in fixed zygotes. The real-time data showed that two microtubule arrays with up to four “poles” form around the two PNi, despite the presence of only two astral MTOCs (i.e., the centrosomes). Moreover, they showed that in living zygotes, centrosomes are not essential for bipolar spindle assembly and that their attachment to the spindle body is rather loose (e.g., allowing the second spindle to capture and remove a centrosome from the first one or a centrosome associating with the spindle midzone rather than the pole). They also showed that centrosomes were lost into the cytosol if localizing more than ∼5 µm away from the PN or spindle body.

### Most spindle microtubules originate from the vicinity of chromosomes

In all live embryos (21/21), the centrosomes nucleated microtubules shortly before and at NEBD. Within 10 min after NEBD, however, the bulk of spindle microtubules seemed to accumulate or even originate in the vicinity of the chromosomes; this was particularly evident when one of the two zygotic spindles was acentrosomal (Fig. 3 B, left PN). Furthermore, centrosomal microtubules, when present, grew preferentially toward the DNA after NEBD and the microtubule signal intensities at centrosomes seemed to decrease already early in mitosis, whereas microtubule mass at the spindle center seemed to increase quickly (Fig. 4 A). To quantify where most of the spindle microtubule mass appeared at different times of early mitosis, we analyzed the changes in spatial distribution of total microtubule intensity over time along the centrosome axis, from prophase to pro-metaphase (Fig. 4, B–D). We were interested in comparing not only the total microtubule mass (Fig. 4 B, black cuboid with solid line) but also concentrations within equally small volumes along the centrosomal axis (Fig. 4 B, black cuboid with dashed line). This analysis revealed that on average, within 5–7.5 min of NEBD, the total microtubule intensity started to increase at the spindle axis center (Fig. 4 C, light dashed line; *n* = 6), and reached a peak of ∼85% by 20 min after NEBD. In comparison, we only observed a modest increase of less than 20% around the centrosomes (Fig. 4 C, dark dashed lines), which peaked ∼5–7.5 min after NEBD. After this initial small increase, the centrosomal microtubule mass declined or stagnated, while the chromosomal microtubule mass continued to rise. Even when comparing the microtubule mass within equal volumes at the axis center (where chromosomes would be located; Fig. 4 D, light dashed line) and at the centrosomes (Fig. 4 D, dark dashed lines), we observed a similar behavior, indicating that microtubule concentration increases more in the vicinity of chromosomes. To analyze and visualize this change in microtubule abundance at the centrosomes and at the chromosomes further, we plotted the change in total mass (Fig. 4 E) and concentration (Fig. 4 F) at both locations over time (*n* = 6). This analysis confirmed that microtubule mass only modestly and transiently increased at the centrosomes until 5–7.5 min after NEBD, whereas chromosomal microtubule mass continued to rise all the way to late pro-metaphase, when spindle assembly was largely complete. At metaphase, the spindles in live zygotes had a barrel-shaped appearance and mostly separated centrosomes (Fig. 3, A–C and Fig. S4, C–E, arrowheads; and Fig. 4 A, pseudo-color profile). Together, these results indicate that chromosomes most strongly contribute to microtubule nucleation and polymerization in the bovine zygote (here, Ran-GTP–dependent pathways; Cavazza and Vernos, 2016), but the Ran-independent chromosome passenger complex can also mediate microtubule nucleation (Maresca et al., 2009; Kelly et al., 2007; Sampath et al., 2004). In addition, the massive increase in microtubule intensity at the spindle midzone could likely be further enhanced by microtubule-dependent microtubule nucleation through the Augmin complex (Petry et al., 2013), which plays a major role in spindle pole organization in acentriolar mouse embryos (Watanabe et al., 2016). However, the Augmin pathway might also be modulated by Ran-GTP–dependent spindle assembly factors like TPX2 in vertebrates (Thawani et al., 2019; Schatz et al., 2003). Centrosomes, on the other hand, seem to contribute little to the increase in overall microtubule mass after NEBD. This is consistent with our observations of few and unstable centrosomal microtubules that make a weak connection to the spindle body in fixed and cold-treated zygotes (Fig. 1 D).

Overall, all our observations in in vitro–generated bovine zygotes are consistent with a model where chromosome-dependent microtubule nucleation pathways and microtubule self-organization are dominant driving forces for bovine zygotic spindle assembly, while the weakly associated centrosomes make only a minor contribution. This explains why pronuclear position and timing of NEBD of the two PNi at the beginning of mitosis are the main determinants of how quickly the two spindles form, align, and merge. It also explains why, when the two PNi are far apart, two spindles are generated that remain separate until chromosome segregation. The fact that spindles form around the chromosomes and incorporate the centrosomes only if they are within ∼5 µm reach of their weak asters also explains the very variable allocation of centrosomes to the four possible spindle poles, with all possible combinations within a bipolar microtubule array, from two centrosomes at one pole to no centrosome at all. This is very different from the situation in somatic cells or *C. elegans* embryos (Müller-Reichert et al., 2010), where the two centrosomes are the dominating centers of microtubule nucleation and thus, from the onset of mitosis, build a single bipolar microtubule array with well-focused poles that captures the chromosomes. Despite the presence of the two sperm centrioles, and eventually two centrosomes, the bovine zygote surprisingly behaves rather similarly to the mouse zygote where the sperm centrioles are degraded. The main difference being that in the mouse, the two spindles cluster some of the many cytoplasmic MTOCs at their poles, whereas in the cow, the two centrosomes are incorporated seemingly randomly only if positioned close by. It will be very interesting in the future to understand the mechanism of chromosomal microtubule nucleation and spindle bipolarization in mouse and bovine zygotes and to carefully compare it to clinical data to infer whether a similar process occurs in human zygotes. Recent studies are pointing toward such a mechanism in human zygotes, especially the observation that the genomes frequently display a multipolar orientation in pro-metaphase and metaphase and that chromosomes are frequently segregated in a uniparental conformation (Ford et al., 2020 *Preprint*).

Different from the mouse, the larger bovine zygotes (∼120 µm in diameter) exhibited a striking degree of incomplete or failed pronuclear migration, which sometimes resulted in very large distances between the two PNi at the onset of mitosis (>30 µm distance). It is important to consider that these events might be particularly prominent in in vitro–fertilized and cultured zygotes. We assured that this phenotype was not caused by defective sperm, as it occurred with gametes obtained from independent bulls. We also validated that standard procedures to remove cumulus cells from fertilized oocytes did not promote defective pronuclear migration. For carrying out experiments in the physiologically valuable bovine model, which shares several similarities with human, we had to rely on oocytes from abattoir cow material and could thus not provide control data from in vivo conceived and developed embryos. The formation of distant dual spindle could be enhanced by the IVF and culture conditions that we have used, which are standard in veterinary and breeding practice. Nonetheless, in vitro–generated bovine embryos are a clinically relevant model for the research of preimplantation mitosis and aneuploidy, as IVF is also standard practice in assisted reproduction treatments for human patients.

The distant PNi in the bovine model provided the opportunity to observe distinguishable dual spindles over a longer time interval without alignment and merging and without centrosomes at both poles, further demonstrating the role of the separate parental genomes for initialization of the dual spindles. It is also important to highlight that while the centrosomes do not seem to be essential for zygotic spindle assembly per se, they may play a role in coordinating pronuclear migration, as has been reported in species such as *C. elegans* (Malone et al., 2003) and very recently in bovine zygotes (Cavazza et al., 2021). In the future, it will be very interesting to investigate the pronuclear migration process further and probe its robustness in mammalian zygotes. The similarities with human zygotes, such as the inheritance of the centrosomes from the sperm and the increased risk of mis-segregation during the early embryotic cleavages, make the bovine zygote a valuable model to study the mechanisms behind the error-prone nature of early embryonic division in nonrodent mammals and have important implications for improving the quality of infertility treatments and better understanding how the parental genomes in the embryo are partitioned and eventually merged.

## Materials and methods

### Bovine oocyte collection, in vitro maturation, fertilization, and zygote culture

Cumulus–oocyte complexes (COCs) were collected from abattoir ovaries and transported to the laboratory within 2 h at 37°C. The ovaries were then washed in physiological saline (0.9% wt/vol NaCl) and briefly stored in physiological saline containing 100 U/ml penicillin and 100 µg/ml streptomycin at 37°C. Follicular fluid and COCs were aspirated from follicles with a diameter of 2–8 mm and collected into 50 ml conical tubes using a 19-gauge needle connected to tubing and a vacuum pump (Ferraz et al., 2018). Only COCs with a minimum of three layers of cumulus cells were selected and washed in Hepes-buffered M199 (22340–020; Gibco-BRL) and then either directly matured in vitro for 23 h in groups of 35–70 COCs in 500 µl of maturation medium (31100–027, NaHCO_3_-buffered M199 [11150059; Gibco-BRL] supplemented with 1% (vol/vol) penicillin-streptomycin [15140122; Gibco-BRL], 0.02 IU/ml Follicle Stimulating Hormone [FSH; Sioux Biochemical], 0.02 IU/ml Luteinizing Hormone [LH; Sioux Biochemical), 7.7 µg/ml cysteamine [30070; Sigma-Aldrich), and 10 ng/ml epidermal growth factor [E4127; Sigma-Aldrich]) at 38°C in a humidified atmosphere at 5% CO_2_ or held at room temperature for 19 h in synthetic oviduct fluid (SOF) for holding (H-SOF; Avantea) before in vitro maturation. After maturation, the oocytes were fertilized using frozen thawed sperm cells from one bull of known fertility. Spermatozoa were selected by centrifugation through a discontinuous Percoll (90/45%; P1644; Sigma-Aldrich) gradient and added at a final concentration of 1 × 106 cells/ml to fertilization medium (Parrish et al., 1988) supplemented with 1.8 IU/ml heparin (H3393; Sigma-Aldrich), 20 µM d-penicillamine (P4875; Sigma-Aldrich), 10 µM hypotaurine (H1384; Sigma-Aldrich), and 1 µM epinephrine (E4250; Sigma-Aldrich). IVF was performed for 6–9 h at 38°C in a humidified atmosphere at 5% CO_2_. Presumptive zygotes were then vortexed for 3 min to remove cumulus cells, transferred to SOF (Takahashi and First, 1992), and cultured at 38°C in a humidified atmosphere at 5% CO_2_ and 5% O_2_. The zygotes used for live imaging were cultured in the same conditions with the absence of Phenol Red in the SOF culture media. On day 5 of culture, cleaved embryos were transferred to a new fresh SOF (500 µl per group of 35–70) and cultured further until day 8 under the above-described conditions.

### Microtubule regrowth after nocodazole treatment

At 27.5 h after fertilization, nocodazole was added to the culture medium of bovine zygotes to reach the final concentration of 5 µM. After 4 h, bovine zygotes were washed three times for 1 min and three times for 5 min in 500 µl ice-cold culture medium. The zygotes were then incubated in warm culture medium (39°C) for microtubule regrowth of different time intervals and then immediately fixed and used for IF imaging as follows.

### IF and confocal imaging

At 27.5 h after fertilization, bovine zygotes were briefly washed in PBS at 38°C and either directly transferred and fixed in 500 µl fixation medium (94 mM Pipes, pH 7.0, 0.94 mM MgCl_2_, 94 µM CaCl_2_, 0.1% Triton X-100, and 1% PFA) or after incubation in ice-cold PBS for 3 min (cold shock treatment) as described for mouse oocytes and embryos (Kitajima et al., 2011; Reichmann et al., 2018b). After 30 min fixation, the embryos were washed four times in 3% BSA in PBS with 0.1% Triton (PBS-T) at 22°C and extracted in PBS-T overnight at 4°C. All the following treatments were done within wells of ibidi µ-Slides (81501, µ-Slide Angiogenesis; ibidi) filled with 40 µl of solution per well. Embryos were blocked in 5% normal goat serum and 3% BSA in PBS-T and then incubated with the primary antibodies in blocking solution overnight at 4°C. The primary antibodies used were chicken anti α-tubulin (10 µg/ml, ab89984; Abcam), rabbit anti-CEP192 (3.5 µg/ml; Ab frontier AR07-PA001), rabbit anti-lamin B2 (4 µg/ml, ab155319; Abcam), rabbit anti-calnexin (4 µg/ml, ab22595; Abcam), mouse anti-acetylated tubulin (5 µg/ml; T7451 Sigma-Aldrich), and mouse anti-NEDD1 (2.5 µg/ml; H00121441-M05, clone 7D10; Abnova). Embryos were then washed three times for 5 min with 3% BSA in PBS-T and incubated with the following DNA dye and secondary antibody dilutions in blocking solution for 3 h at 22°C: Hoechst 33342 (0.2 mM; Sigma-Aldrich), goat anti-chicken Alexa Fluor 647 (4 µg/ml, A-21449; Molecular Probes), goat anti-mouse Alexa Fluor 488 antibody (8 µg/ml, A-11029; Invitrogen), and goat anti-rabbit Alexa Fluor 568 antibody (8 µg/ml, A-11036; Invitrogen). The embryos were then washed three times for 10 min with 3% BSA in PBS-T and two times for 10 min with PBS alone and mounted on glass slides (Superfrost Plus; Menzel) with anti-fade mounting medium (Vectashield; Vector Laboratories).

Fixed bovine zygotes were imaged at 20–22°C using a Leica SPE-II– DMI4000, 1X PMT spectral detector equipped with a 63× oil-immersion objective (1.3 numerical aperture). Z-stacks of ∼80 µm were acquired at 42.7 nm in xy and 420 nm in z. Staining of Cep192 lead to high background noise. The specific staining was therefore validated by colocalization of the α-tubulin staining or, in case of cold-treated zygotes, it was replaced with NEDD1, which showed minimum background staining. To exclude that dual spindles resulted from polyspermy, we stained for acetylated tubulin of the residual sperm flagellum and only documented and analyzed embryos with a single flagellum (Fig. S1 A) or scored for diploidy comparing the volumes of segmented DNA.

### Expression constructs and mRNA synthesis

Constructs used in this study to synthesize mRNA, pGEMHE-H2B-mCherry (Kitajima et al., 2011), pGEMHE-EGFP-MAP4 (Schuh and Ellenberg, 2007), were previously described. To generate pGEMHE-EB3-EGFP2, full-length *Homo sapiens* EB3 cDNA (NM_001303050.1, a generous gift from Niels Galjart, Department of Cell Biology, Erasmus Medical Center, Rotterdam, the Netherlands) was tagged at the C terminus with a tandem mEGFP and cloned into the vector pGEMHE with a T7 promotor sequence for mRNA production. From linearized template DNA (1 µg), capped and poly-adenylated mRNA was synthesized in vitro using the mMESSAGE mMACHINE T7 ULTRA Transcription kit (AM1345; Thermo Fisher Scientific). The mRNA was purified (74104, RNeasy Mini Kit; QIAGEN) and dissolved in 14 µl RNase-free water.

### Micromanipulation

Zygotes were sorted apart from unfertilized oocytes through scoring for two polar bodies. The cow zygotes were then injected with mRNA in solution as described for mouse oocytes (Schuh and Ellenberg, 2007; Jaffe and Terasaki, 2004), with some modifications. In brief, an “injection slit” was created between the edges of two glass coverslips by using a spacer of two layers of double-sided adhesive tape (05338; tesa) tightly pressed together (∼180 µm) to accommodate the cow zygotes of ∼120 µm in diameter. The tape was glued 2 mm from the edge of one coverslip and the second coverslip attached with ∼300 µm overhang. Using silicone grease, an intact coverslip was attached to a U-shaped support slide and the “coverslip–tape sandwich” was also attached on the other side, the injection slit facing toward the opposing intact coverslip and thus toward the inside of the formed chamber. The whole chamber was then filled with 37–38°C warm MOPS buffer before pipetting the embryos into the slit, but during injection (∼20 min), the temperature in the room was ∼23°C. Injection needles with appropriate tip diameter were generated using a P-1000 Flaming/Brown micropipette puller (Sutter Instruments) with a square box filament (heat, 497; pull, 30; velocity, 120; time, 200; pressure, 250; ramp, 497). The injection volume (4–5 pl) was adjusted to ∼0.5% of the bovine zygotic volume. The mRNA concentrations ranged between 0.1 and 0.2 µg/µl for H2B-mCherry, 0.5 and 0.9 µg/µl for EB3-mEGFP2, and 0.3 and 0.4 µg/µl for MAP4-EGFP.

### Live imaging

For time-lapse imaging of cow zygotes, the in-house–built inverted light-sheet microscope was used (Strnad et al., 2016; Reichmann et al., 2018a) with minor additional modifications. In brief, using silicone glue (Silpuran 4200; Wacker), we assembled a 25-µm-thick fluorinated ethylene propylene (FEP) foil (RD-FEP100A-610; Lohmann) into a boat-shaped sample holder made of polyether ether ketone (PEEK) that has an opening slit at the bottom, where the foil with the same refractive index as water creates a transparent base for sample mounting. In the inverted microscope setup, two water-dipping objectives facing upwards allow sample mounting according to standard microdrop in vitro embryo culture, as well as imaging of several embryos in parallel. To position the bovine zygotes on the FEP foil, the transparent SOF culture medium (150–200 µl, 38°C, 5% CO_2_) was pipetted onto the foil base in the holder and covered with Ovoil (750 µl, 10029; Vitrolife) at the same temperature and gas conditions to avoid evaporation. Then, pockets were stamped into the covered foil using a bulb-tipped glass capillary with ∼150 µm diameter (as illustrated in Reichmann et al., 2018a) and the embryos transferred into these pockets within SOF medium one by one. Imaging was also performed at 38°C, 5% CO_2_, and 5% O_2_. In the microscope setup, the scanned laser beam is directed through the illuminating lens pair of a 10× water-immersion objective lens with a numerical aperture of 0.3 (CFI Plan Fluor 10XW; Nikon) and a tube lens (f = 200 mm; Nikon) creating the illumination sheet. The emitted fluorescence is collected by the orthogonally placed 100× detection objective with a numerical aperture of 1.1 (CFI Plan 100XW; Nikon). A tube lens (f = 200 mm; Nikon) generates the image at an intermediate image plane where a circular aperture is placed to limit the field of view to 130 µm. Thereafter, the image is demagnified four times using two relay lenses (f = 300 mm, 49–280-INK, and f = 75 mm, 47–639-INK; Edmund Optics) and the image is detected on a scientific complementary metal-oxide semiconductor camera (Neo sCMOS; Andor) with a resulting pixel size of 130 nm in xy. For imaging of chromatin and either microtubule tips or lattice, fluorescence from H2B-mCherry and either EB3-mEGFP2 or EGFP-MAP4 was acquired simultaneously every 2.5 min using a 488-nm laser (∼25–30 µW) and a 561 nm laser (∼5–10 µW) with an exposure time of 100 ms. Stacks of 100–104 µm were acquired by 101 planes, resulting in a *z*-step size of 1–1.04 µm. As described by Strnad et al. (2016), an in-house–developed LabVIEW program (National Instruments) was used to control the microscope. Timing of laser intensities, galvanometric scanner positions, and camera acquisition were ensured by a custom-written program implemented in Field Programmable Gate Array (FPGA) module (NI PCIe-7841R; National Instruments).

### Raw image processing

Time-lapse images were processed to extract single color data from the raw camera data as described originally for the iSPIM (inverted Selective Plane Illumination Microscopy) data (Strnad et al., 2016). We also used Fiji (Schindelin et al., 2012) with a new in-house built plugin for visualizing and initial processing of large image data using lazy loading (Tischer et al., 2020 *Preprint*). This application was used for channel splitting and composing, image cropping, and maximum intensity projecting. For the live data, subsequent volume rendering (Fig. 3; Fig. S4, C–F; and Fig. S6), and movie generation (Video 1, Video 2, Video 3, Video 4, and Video 5) was performed using arivis Vision4D release 3.1–3.3. Scale bars were calculated manually for releases 3.1–3.2 and automatically annotated in release 3.3.

For the IF data the volume rendering of the confocal z-stack was obtained with the 3D view of Imaris 8.1 (Bitplane) and the maximum projection perpendicular to the spindle axis was acquired with the snapshot option.

### Quantification of α-tubulin IF intensity for spindle half comparison

An in-house–developed MATLAB (MathWorks) script was used to quantify IF intensity from α-tubulin staining and perform a robust comparison of the intensity distributions of one spindle half with respect to the other. The script first segmented the signal from the metaphase chromosomes from the separate Hoechst channel and predicted the orthogonal spindle axis from the shape of the metaphase chromosomes. It then generated a set of parallel and equidistant cross sections of the tubulin channel orthogonal to the predicted axis.

To segment chromosomes, the Hoechst channel was first interpolated along the z direction to generate an isotropic 3D stack from anisotropic raw data. A 3D Gaussian filter was applied on the interpolated stack to reduce the noise where sigma and kernel size of the filter were set to 2 and 3, respectively. The Hoechst channel was binarized by combining parameters from adaptive thresholding (Otsu, 1979) applied on individual xy planes of a z-stack, as well as on all xy planes of the stack together (Hériché et al., 2014). The chromosome mass was identified by connected component analysis of the detected binary objects followed by smoothing operations. The spindle region was also detected using a similar approach, while centrosome coordinates were picked manually. 3D coordinates of all the voxels belonging to the detected chromosome mass were used to construct a Hessian matrix. The eigenvector with the lowest eigenvalue of this matrix approximates an orthogonal vector to the metaphase plate and thus was taken as the predicted spindle axis.

The predicted spindle axis was used as a reference to slice the microtubule channel at 500 nm spacing, generating a set of parallel cross sections orthogonal to this axis. The slicing procedure was described in detail in Walther et al. (2018). In brief, a total of 24 µm in length along the predicted axis (12 µm in each direction from the centroid of chromosome mass) was taken for slicing. The size of a slice was 621 × 621 pixels, where the cross section with the predicted spindle axis defined the center of the slice. To quantify microtubule intensity, the average background intensity was estimated first and was subtracted from the tubulin channel before the slicing. The average background intensity was calculated from a rim of 2 pixels in width at 8 pixels from the boundary of the segmented spindle region. The total background subtracted intensity of tubulin inside each slice was plotted with respect to its distance from the centroid of the chromosome mass (see Fig. 2 B; and Fig. S4, A and B). This intensity profile was further analyzed to extract different parameters to describe the shape and intensity of two spindle halves.

The valley between two intensity peaks from the profile was detected first to define parts of the profile belonging to individual spindle halves. Total intensity of a spindle half was calculated by summing up the AUC belonging to that spindle half. To estimate the width of intensity distribution belonging to a spindle half, the intensity at the valley was subtracted from the profile (opaque area under distribution valley, Fig. 2 B). FWHM of the valley subtracted intensity distribution from each spindle half were calculated. Both parameters (total intensities and FWHMs of spindle halves) were normalized to either the acentrosomal half (for monocentrosomal spindles) or the half with the smaller measure (for bicentrosomal spindles; Fig. 2, C and D).

The length of a spindle half was also calculated from the respective part of the original intensity profile (without subtracting the intensity at the valley). The distance between the valley and the other edge of the intensity distribution, representing the slice at the periphery of a spindle half, was used to determine the length. The “polar” periphery of each spindle half was detected by taking the peripheral position closest to the peak of the profile where intensity value was less than 10% of the peak. If no such slice was found (all values are higher than 10% of the maximum, *n* = 1), the furthest slice from the peak was considered as periphery of a half. The lengths of the spindle halves were also normalized to either the acentrosomal half (for monocentrosomal spindles) or the shorter half (for bicentrosomal spindles; Fig. 2 E).

### Quantification of microtubule regrowth at centrosomes and chromosomes

To compare the mass of microtubules at the centrosome and chromosomes from IF data (Fig. S5 C), we first manually segmented genomic and centrosomal volumes in the respective Hoechst and pericentrosome channels and imaging volumes using arivis Vision4D software. Where the parental genomes were still separate, one parental genome and one (peri)centrosome at large distance were segmented per zygote; otherwise, the whole genomic volume within a 3D stack was segmented. We then smoothened the α-tubulin IF using a median-based denoising filter (diameter = 0.5 µm) and finally segmented the α-tubulin signal in the respective genomic and centrosomal volumes applying threshold segmentation in the intensity range of 30–255 of the 8-bit images. From these segments, we calculated the intensity sum per positive voxel counts and plotted them ratiometrically.

### Calculating distances from centrosomes to the spindle body and to chromosomes

To calculate the distance between centrosome and spindle body from IF data (d_1_; Fig. S1 E), the axis between the centrosome and chromosome centroid was used as a reference for slicing the microtubule channel (slicing described in previous section). In this case, slicing along this axis was performed at 200 nm spacing starting from the centrosome toward the chromosome centroid. The total intensity of each slice was calculated to create an intensity profile along the axis. The maximum and minimum intensity values were determined first, and the minimum intensity was subtracted from the profile. The minimum subtracted profile was probed starting from the centrosomal end. The last location in the profile, where the total intensity was less than 10% of the maximum total intensity, was determined as the periphery of the spindle body. The distances between the manually annotated centrosome coordinates and the determined spindle body (d_1_) or the chromosome centroid (d_3_) and between the chromosome centroid and the spindle body (d_2_; Fig. S1 E) were then calculated (Fig. S1, F and H).

This centrosome–spindle distance (d_1_; Fig. S1 E) was normalized to the length of the respective spindle half (d_2_) to illustrate distance relations (d_1_/d_2_; Fig. S1 G).

### Quantification of dynamic microtubule distribution of EGFP-MAP4 and EB3-mEGFP2 signal

Microtubule signal intensity (EGFP-MAP4 or EB3-mEGFP2) was quantified along the centrosomal axis defined by the two centrosomes. The center of intensities and thus central coordinates of the centrosomes were determined from manually segmented microtubule signal at centrosomes using arivis Vision4D. Original anisotropic stacks were first interpolated along the z direction to create isotropic 3D stacks, and a microtubule intensity profile from one centrosome to the other was generated. For each time point, a total of 15 equidistant parallel slices starting from one centrosome to the other were generated. The slices were taken orthogonal to the centrosomal axis where the center of each individual slice was intersected by the axis. The size of a slice was set to either 351 × 351 pixels (black cuboid with solid line, Fig. 4 B) to determine the total microtubule mass along the centrosomal axis and within the entire spindle at a given time (Fig. 4 C), or the slice size was set to 15 × 15 pixels (black cuboid with dashed line, Fig. 4 B) to estimate the microtubule concentration along the centrosomal axis (Fig. 4 D). The distance between two centrosomes was variable in different time points within a zygote as well as between zygotes. To address this, a fixed number of slices between two centrosomes was generated. This normalized the distances between two consecutive slices in different stacks with respect to the distance between centrosomes. The total intensity of each slice was calculated and normalized to the maximum total intensity considering all slices and all time points within a zygote. Normalization of intensity and interslice distance made the extracted intensity profile comparable within a zygote as well as between different zygotes. This allowed computation of an average intensity profile (Fig. 4, C and D) over time and intensity change over time at different landmarks (such as centrosomes and the center of the spindle; see Fig. 4, E and F) using the data from all the analyzed zygotes (*n* = 6).

### Data transformation into 2D sections parallel to the centrosomal axis

To display the kinetics of microtubule intensity over time (Fig. 4 A), 3D data were transformed and resliced orthogonal to the centrosomal axis so that both centrosomes were visible in the same 2D slice. The raw data were interpolated as described in the previous section to generate an isotropic 3D stack. The interpolated data were then translated in xy so that the midpoint between two centrosomes moved to the center of the translated image. The angle between centrosomal axis (defined by the two centrosomes) and the xy plane was calculated and the translated stack was rotated to align the centrosomal axis to the xy plane. The angle between centrosomal axis and x axis was calculated and the data were further rotated to align the centrosomal axis to the original x axis. Bicubic interpolation was used during the rotation. All the data were transformed in the same way so that the kinetics of microtubule intensity at centrosomes as well as the center of the centrosome axis could be observed in the same 2D slice over time.

### Statistical analyses

Bar and dot plots were generated using GraphPad Prism. Bars represent means ± SEM. Statistical significance was determined by unpaired two-tailed Student’s *t *tests. P values <0.05 were considered statistically significant. Data distribution was assumed to be normal, but this was not formally tested.

### Online supplemental material

Fig. S1 shows IF data illustrating diverse mis-localizations of centrosomes to spindles poles after monospermic fertilization and additional quantitative data supporting results shown in Fig. 1. Fig. S2 shows IF staining for nuclear lamina throughout zygotic mitosis, Fig. S3 the equivalent for the endoplasmic reticulum. Fig. S4 shows microtubule distributions along the spindle axis in mono- versus bicentrosomal spindles from IF images supporting Fig. 2 and single-channel live-imaging data from composites displayed in Fig. 3. Fig. S5 displays the extent of microtubule regrowth at centrosomes and around chromosomes after nocodazole washout. Fig. S6 shows live-imaging data of five mitotic zygotes highlighting diverse centrosome positioning in dual spindle assembly supporting results from Fig. 1 and Fig. 3. Video 1, Video 2, Video 3, Video 4, and Video 5 show dual spindle assembly in three different zygotes. Video 1 corresponds to the selected time points in Figs. 3 A and S4 C. Video 2 corresponds to Figs. 3 B and S4 D. Video 3, Video 4, and Video 5 represent the same zygote with distant dual spindles around the two distant PNi, also shown in Fig. 3 C and Fig. S4, E and F. Video 3 and Video 4 show the individual spindles (individual pronuclear volumes). Video 5 shows both spindles in the zygotic volume.
